# Severe Malaria with a Rare Tetrad of Blackwater Fever, Acute Renal Failure, Disseminated Intravascular Coagulopathy, and Acute Acalculous Cholecystitis

**DOI:** 10.1155/2023/5796881

**Published:** 2023-05-02

**Authors:** Hira Hanif, Biraj Shrestha, Salina Munankami, Manish Shrestha, Bidhya Poudel, Roopika Reddy, Syed Jaleel, Debra Powell

**Affiliations:** ^1^Reading Hospital Tower Health, West Reading, PA, USA; ^2^Khatmandu Medical College, Kathmandu, Nepal; ^3^Amita Health St Francis Hospital, Evanston, IL, USA

## Abstract

**Background:**

Blackwater fever (BWF) is a severe clinical syndrome occurring as a complication of malarial infection characterized by intravascular hemolysis, hemoglobinuria, and acute renal failure in people exposed to *Plasmodium falciparum* and, to some extent, in people who were exposed to medications like quinine and mefloquine. The exact pathogenesis of classic BWF remains unclear. The mechanism leading to damage to the red blood cells (RBCs) can be immunologic nonimmunologic, leading to massive intravascular hemolysis. *Case Presentation*. We present a case of classic blackwater fever in a 24-year-old otherwise previously healthy male without any history of antimalarial prophylaxis use, returning from recent travel to Sierra Leone. He was detected to have *P. falciparum* malaria in the peripheral smear test. He was treated with artemether/lumefantrine combination therapy. Unfortunately, his presentation was complicated by renal failure and was managed with plasmapheresis and renal replacement therapy.

**Conclusion:**

Malaria continues to be a parasitic disease that can have devastating effects and continues to be a challenge globally. Although cases of malaria in the United States are rare and cases of severe malaria, mainly attributed to *P. falciparum*, are even more uncommon. Care should be taken to retain a high level of suspicion to consider the diagnosis, especially in returning travelers from endemic areas.

## 1. Introduction

Malaria is the infection caused by obligate intraerythrocytic protozoa of the genus *Plasmodium* is a rare disease in the United States, with most cases imported from travel to endemic malarial regions [1]. According to the 2022 WHO malaria report, globally there were an estimated 247 million malaria cases in 2021 in 84 malaria endemic countries (including the territory of French Guiana), an increase from 245 million in 2020, with most of this increase coming from countries in the WHO African Region [2]. In 2015, the baseline year of the Global Technical Strategy for malaria 2016–2030 (GTS), there were an estimated 230 million malaria cases [2].

Severe malaria is defined as the presence of *P. falciparum* malaria and any one of the following: *Plasmodium falciparum* parasitemia >10%, signs of organ dysfunction, including renal impairment (creatinine > 3 mg/dl), severe anemia (hemoglobin <6 g/dl), recurrent bleeding and bleeding disorders, acidosis, impaired consciousness, convulsions with two or more episodes in 24 hours, hypoglycemia (<40 mg/dl), pulmonary edema, and shock [1–4]. Some other rare but serious presentations of malaria are blackwater fever, disseminated intravascular coagulation (DIC), and malarial acute acalculous cholecystitis (AAC) [1, 3–6]. We present an interesting case of severe malaria in the United States who unfortunately had all three rare complications of severe malaria with an excellent response to artesunate.

## 2. Case Presentation

We are reporting the case of a previously healthy 24-year-old African American male with no significant past medical history who presented to the emergency department with six days of fever, headache, fatigue, and myalgia. Travel history was notable for a 3-week trip to Sierra Leone, West Africa, from where he returned one week before the presentation. He was in his usual state of health before and during his trip and denied insect bites or sick contacts. In addition, he denied any history of immunodeficiency, splenectomy, or hematologic disorder. A review of systems was negative for diarrhea, dysuria, cough, runny nose, neck rigidity, nausea, or vomiting. He did not receive pretravel prophylaxis against malaria, hepatitis A, or yellow fever. On examination, he was ill-appearing and lethargic but oriented to time, place, and person. On abdominal examination, he had epigastric and right upper quadrant tenderness. The rest of the physical examination was unremarkable.

**Figure 1:**
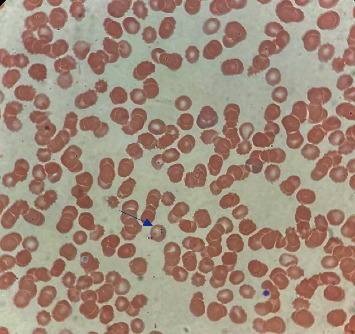
Peripheral blood smear showing trophozoite in various stages of development with an arrow pointing towards signet-ring classically seen in *Plasmodium falciparum* infection.

On the day of admission, laboratory studies showed a white cell count 6000/ml, platelets 41,000/ml, hemoglobin 13.4 g/dl, alanine transaminase 56 U/l, aspartate transaminase 103 U/l, total bilirubin 8.4 mg/dl, and serum creatinine 1.02 mg/dl (Table 1). The acute hepatitis panel was negative. A blood smear revealed ring-shaped trophozoites consistent with *Plasmodium falciparum* with a blood parasitemia percentage of 1% (Figure 1). He was initiated on treatment with hydroxychloroquine that was switched to atovaquone-proguanil after one dose due to the high probability of chloroquine-resistant *P. falciparum* in the endemic region of Africa as per infectious disease recommendation. Imaging with a CT scan of the abdomen did not show any abnormality.

### 2.1. On Day 2

The patient's condition worsened; he reported feeling worse than the day before. He met the criteria for severe malaria based on a parasitemia percentage of 1.7%, a plasma lactate level of 6.3 mmol/L, and an elevated serum total bilirubin of 7.5 mg/dL. He was then planned to be switched to intravenous artesunate, but unfortunately, it was not available in the health system, and CDC was contacted to obtain supplies. In the interim, he was started on quinine 648 mg per oral every eight hours, along with doxycycline 100 mg per oral dose twice daily. Later in the evening of the 2^nd^ day, the patient's condition further deteriorated along with his labs: lactic acid rose to 8.6 mmol/L, creatinine levels rose to 1.56 mg/dL, and parasitemia percentage increased to 9%. A complete blood count showed a further decrease in white cell count to 3600/ml, hemoglobin of 11.3 g/dl, platelet of 20,000/ml, and d-dimer more than 20 mg/l, fibrin split products more than 40, fibrinogen 375 mg/dl, and an INR of 1.6 (Table 1). His ISTH criteria score was six, compatible with overt disseminated intravascular coagulation (DIC). Hematology was consulted, and the patient was shifted to the medical ICU for closure monitoring.

### 2.2. On Day 3

Artesunate was procured early, and the patient received the first dose around 4 am. The patient complained of a worsening abdomen, and the abdominal examination was positive for Murphy's sign. We then got an abdominal ultrasound, which showed extensive gallbladder wall thickening with no stones. Furthermore, a HIDA scan did not reveal gallbladder sludge or stone with delayed emptying of the gallbladder after 2 hours. This confirmed that he had developed acalculous cholecystitis. Given his critical illness with very low platelets and the HIDA scan showing delayed emptying of the gallbladder, we decided to clinically monitor him with no surgical intervention. He also had started to show signs of kidney failure with fluid overload, decreased urine output, and rising creatinine levels (Table 1).

### 2.3. On Day 4

The next day the patient's parasite percentage had decreased to 3%. Artesunate therapy was continued. He complained of shortness of breath, with examination showing bibasilar crackles and peripheral edema suggesting fluid overload. The labs showed that renal function had further declined (Table 1). We gave him a trial of intravenous furosemide; however, his urine output did not improve, so nephrology was consulted. Given the progressive renal dysfunction, presumably secondary to free hemoglobin-induced renal tubular damage, plasmapheresis seemed an appropriate modality to remove free hemoglobin and reduce continued tubular damage. A temporary right internal jugular vein dialysis catheter was placed, and the patient underwent plasma exchange followed by hemodialysis.

### 2.4. On Day 5

His parasite count had dropped to 0.5%. He was switched to artemether-lumefantrine therapy. He was continued on plasma exchange and hemodialysis.

### 2.5. On Day 10

Even though the patient's creatinine remained elevated at 9.14 mg/dl, his urine output had improved, and he remained euvolemic on an exam, despite not receiving dialysis for three days, so further hemodialysis was not done. In addition, all his other labs had significantly improved (Table 1). He was safely discharged home with outpatient follow-up with infectious disease and nephrology.

## 3. Discussion

Blackwater fever (BWF), inspired by the French word “fievre bilieuse melanurique,” is a severe clinical syndrome occurring as a complication of malarial infection characterized by intravascular hemolysis, hemoglobinuria, and acute renal failure in people exposed to *P. falciparum* [3, 4, 7–9]. It is mainly associated with disease from *P. falciparum* like in our case, but has also been documented in *Plasmodium vivax*, *Plasmodium malariae*, and mixed infections [4, 7, 10]. *Plasmodium falciparum* Thirty percent of severe malaria cases are seen in patients with *P. falciparum* malaria [11–13]. The vector for *Plasmodium* spp. is a female Anopheles mosquito that inoculates sporozoites contained in her salivary glands into the puncture wound when feeding [14]. Apart from the above, other host factors involved in BWF are glucose-6-phosphate dehydrogenase (G6PD) deficiency and exposure to aminoalcohol antimalarial prophylaxis medications like quinine, mefloquine, and halofantrine, and/or concurrent infection with other viruses and bacteria [3, 4, 7–10].

The exact pathogenesis of classic BWF remains unclear; however, some postulated mechanisms leading to damage to the red blood cells (RBCs) are immunologic and nonimmunologic, leading to massive intravascular hemolysis [8]. Antibodies in the immunologic mechanism directed against the RBCs require the presence of a particular compound to interact. The amount of hemolysis thus depends on the affinity of the antibodies binding to the RBC (quantity, specificity, temperature range, ability to fix tissue, and macrophages) as well as those of the target antigen (density, expression, and the patient age). Intravascular hemolysis occurs when IgM antibodies activate the classical complement pathway leading to cell lysis [8]. Another critical factor predisposing BWF reported in many studies is exposure to quinine which may have significant metabolic components for the causation [15]. Studies have suggested that the metabolism of quinine by the cytochrome P450 3A4 enzyme may be responsible for increased oxidative stress within the erythrocytes making the cells more vulnerable to hemolysis in those with malaria and/or G6PD deficiency and thus causing BWF [10, 15]. This has been increasingly reported likely because of the increasing prophylactic/therapeutic use of quinine and mefloquine due to developing resistance of *P. falciparum* malaria to other antimalarial drugs [3, 8–10]. Literature review supports that cases had disappeared mainly after the availability of chloroquine as 1^st^ line treatment replacing quinine during the 2^nd^ World War and that patients showed resurgence in the 1990s after quinine and other amino-alcohol drugs resurfaced in response to chloroquine resistance [3, 4, 9]. Such incidences with other medications containing artemisins, chloroquine, or piperaquine cause oxidant hemolysis are much lower and have rarely been reported. Thus, once BMW is diagnosed, the presumed trigger should be withdrawn immediately with the continuation of antimalarial treatment for patients with high parasitemia. Appropriate antimalarial agents in such a situation would be artemisin derivatives like in our patient, atovaquone-proguanil, or sulphadoxine-pyrimethamine [3].

Acute renal failure (ARF) has been reported in severe forms of malaria, such as blackwater fever (BWF), which is associated with high mortality rates of 15–45% if not managed promptly and correctly [8]. Acute renal failure (ARF) has been reported in severe forms of malaria, such as blackwater fever (BWF), which is associated with high mortality rates of 15–45% if not managed promptly and correctly [8]. The clinical pathophysiology of malaria ARF is complex and multifactorial, including volume depletion, hypoxia, shock, hyperbilirubinemia, intravascular hemolysis, hyperparasitemia, and other factors [6–9]. Acute renal failure has the clinical and biochemical features of ischemic acute tubular necrosis (ATN). It is the most common cause resulting from hypovolemia, peripheral pooling of blood, and the blockage of microcirculation by the parasitized RBCs [8]. Oliguria/anuria, liver dysfunction, jaundice, anemia, thrombocytopenia, proteinuria, hyponatremia, hyperkalemia, and hemoglobinuria have all been reported. In BWF, most people have been noted to die from complications related to acute renal failure. Thus, it is imperative to correct the fluid and electrolyte abnormality since dehydration is the cause of the rapid onset of renal failure [8]. In severe malarial renal failure cases, renal replacement therapy improves the prognosis. Peritoneal dialysis is more effective than hemodialysis in some studies [8]. No mortality was reported among the patients who did not require dialysis and were conservatively treated [8].

Apart from the above complications, different malarial gastrointestinal complications have been reported in other studies, one of which is acute acalculous cholecystitis (AAC), as seen in our patient [5]. It is said to be extremely rare, with only a few reported cases [5, 6]. The first reported case of ACC associated with malaria was in 1999 [6]. The lack of the case is possible because of underreporting since signs and symptoms of AAC are nonspecific, which could be just similar to symptoms of malaria like abdominal pain, diarrhea, and vomiting [5, 6]. However, other symptoms more specific to cholecystitis are tenderness in the right hypochondrium, positive Murphy's sign, jaundice, and dark urine, which should be looked for in cases of suspicion. The pathophysiology of acalculous cholecystitis in association with malaria is multifactorial. The primary mechanism of this illness is thought to be bile stasis resulting from increased bile viscosity and impaired gallbladder contraction [5]. Patients with malaria are more predisposed to increased bile viscosity due to fever and dehydration, leading to a decrease or absence of cholecystokinin-induced gallbladder contraction [5]. Apart from the above, gallbladder wall ischemia that occurs because of a low flow state due to dehydration, hypotension, sequestration of parasites in the gallbladder microvasculature, and adherence of parasitized red blood cells to the vascular endothelium causing microvascular obstruction and ischemia in multiple organs play a role in the pathogenesis of acalculous cholecystitis [5, 6]. The other mechanism is the release of proinflammatory cytokines (TNF, IL-6, IL-8, IL-12, or IL-18) during malarial infection, which can contribute to gallbladder inflammation. These phenomena have been related to complications like brain injury and acute tubular necrosis causing acute kidney injury. Abdominal ultrasonography is the initial imaging study in the evaluation of suspected AAC. The criteria used for diagnosis are gallbladder wall thickening >3 mm, distension of gallbladder, the ultrasonographic Murphy's sign, and the presence of pericholecystic fluid and sludge in the absence of stones [5, 6]. The combination of two or more of the abovementioned criteria and a characteristic clinical picture is considered diagnostic. Although AAC is classically associated with many life-threatening complications, it has not been described with malarial AAC [5]. Care should still be taken to avoid the complications since they cannot be ruled out. Although no definitive management strategies are available for malarial ACC, continuing malarial treatment without any surgical intervention looks promising, as described in different case reports, including our case [5, 6]. Thus, unless there are complications such as perforation or gangrene of the gallbladder, the treatment of malarial AAC is conservative with intravenous fluids and malarial medications with or without antibiotics [5, 6].

## 4. Conclusions

Malaria continues to be a parasitic disease that can have devastating effects and continues to be a challenge globally. Although cases of malaria in the United States are rare, and cases of severe malaria, mainly attributed to *P. falciparum*, are even more uncommon, care should be taken to retain a high level of suspicion to consider the diagnosis, especially in people returning from recent trips and prolonged stays in endemic areas. Physicians need to be aware of the various rare and severe presentations of malarial disease to ensure prompt diagnosis and appropriate treatment, especially since the treatment is particular [3, 7, 8]. Malaria prevention, early diagnosis, and prompt treatment are the only measures to prevent BMF, ARF, and other fatal outcomes [7, 10].

## Figures and Tables

**Table 1:** Laboratory findings.

	Day 1	Day 2	Day 3	Day 4	Day 5	Day 6	Day 7	Day 10	Reference range
Parasitemia %	1%	9%	7.6%	3%	0.5%	None	None	None	None
Medication	AP^*∗*^	Q + D	Artesunate		Artemether/lumefantrine				
Creatinine	1.02	0.98	3.34	5.43	7.05	8.71	8.66	8.80	0.60–1.30 mg/dL
Hemoglobin	13.4	12.4	10.9	10.2	10	9.3	8.7	8.8	14.0–17.5 g/dL
WBC	6.0	2.5	4.7	7.0	8.7	10.1	11.3	7.2	4.8–10.8 × 10^3^/*μ*L
Platelet	41	22	18	28	50	58	108	462	130–300 × 10^3^/*μ*L
Haptoglobin	NA	<30	<30	NA	NA	NA	NA	<30	44–215 mg/dL
LD	NA	525	1664	1005	974	577	503	523	140–271 IU/L
Total bilirubin	8.4	7.5	18.6	9.3	8.1	3.7	2.4	1.9	0.3–1.0 mg/dL
Direct bilirubin	3.9	3.6	14	5.7	4.8	2.3	1.4	0.8	0.0–0.2 mg/dL
AST	103	121	314	172	201	106	94	55	13–39 IU/L
ALT	56	59	73	68	81	57	47	60	7–52 IU/L
D dimer	NA	>20	>20	17.33	11.70	3.26	1.68	NA	<0.50 *μ*g/mL
Fibrinogen	NA	375	415	428	428	399	385	NA	222–525 mg/dL
Fibrin split products	NA	>40	>40	NA	10–40	10–40	10–40	NA	<10 *μ*g/mL
Prothrombin time	NA	18.0	17.6	14.3	13.2	14.1	13.8	NA	11.7–14.2 seconds
INR	NA	1.6	1.5	1.1	1.0	1.1	1.1	NA	0.9–1.1
PTT	NA	40.5	38.3	31.2	29.3	30.2	30.3	NA	23.1–34.3 seconds
Lactic acid	NA	6.3	4.6	NA	NA	NA	NA	NA	0.6–1.4 mmol/L

LD: lactate dehydrogenase, AST: aspartate transaminase, ALT: alanine transferase, PTT: partial thromboplastin, AP^*∗*^: atovaquone proguanil, Q + D: quinine + doxycycline.
